# *Australobiustracheoperspicuus* sp. n., the first subterranean species of centipede from southern China (Lithobiomorpha, Lithobiidae)

**DOI:** 10.3897/zookeys.795.28036

**Published:** 2018-11-08

**Authors:** Qing Li, Su-jian Pei, Xuan Guo, Hui-qin Ma, Hui-ming Chen

**Affiliations:** 1 Guizhou Institute of Biology, Guiyang, Guizhou 550001, PR China; 2 School of Life Sciences, Hengshui University, Hengshui, Hebei 053000, PR China; 3 Scientific Research Office, Hengshui University, Hengshui, Hebei 053000, PR China

**Keywords:** *
Australobius
*, cave Lithobiomorpha, China, new species

## Abstract

*Australobiustracheoperspicuus***sp. n.** (Lithobiomorpha: Lithobiidae) was recently discovered from the Cave of the brickyard of Gaofeng village, in the Guizhou Province, southwest China, and it is described here. Morphologically the new species is similar to *A.magnus* (Trozina>, 1894) from north-western China. The new species can be easily distinguished from those by the trachea connected to the valve of the TIII clearly visible from the dorsal side, the absence of ocelli on each side of the cephalic plate, the DaC spine being only present on the XIII^th^–XV^th^ legs. Numbers of examined specimens, distribution and the main morphological characters and an identification key to the known Chinese species of genus *Australobius* based on adult specimens is given.

## Introduction

The World Catalogue of Centipedes (Bonato et al. 2016) currently records 33 species/subspecies for the genus *Australobius* Chamberlin, 1920, mostly in South-East Asia and East Australia (Zapparoli and Edgecombe 2011). The genus is characterized by possessing the following traits (Eason 1978, 1997, Edgecombe 2002, Zapparoli and Edgecombe 2011): antenna mostly with 20 articles, some species more than 24; ocelli few (e.g., 1+3–1+6), some species more than 8; forcipular coxosternal teeth at least 3+3; tergites with more or less distinct posterior triangular projections; female gonopods with uni-, bi- or tridentate claw, 3+3–4+4 spurs, rarely more than 4; tarsal articulation of legs I–XIII indistinct in some species.

The myriapoda fauna of China is still poorly known and this is especially the case with centipedes of the order Lithobiomorpha. Only 80 species/subspecies of lithobiomorphs are to date known from the country (Ma et al. 2014a, b, 2015, 2018; Pei et al. 2014, 2015, 2016, 2018; Qin et al. 2014, 2017; Qiao et al. 2018; Chao et al. 2018). Altogether, only five species of *Australobius* have been recorded from China (Ma et al. 2008a, b, 2014b, Qin et al. 2014). However, none of them have been recorded from subterranean environment. The present note is devoted to the description of a new cave-dwelling *Australobius* from southern China, as well as the first subterranean species of Lithobiomorpha from China. An identification key is also given to all six species of *Australobius* known to occur in China.

## Materials and methods

All specimens were hand-collected in cave and preserved in 75 % ethanol. Illustrations and measurements were produced using a ZEISS SteREO Discovery. V20 microscope equipped with an Abbe drawing tube and an ocular micrometer and axiocam 512 color. The colour description is based on specimens fixed in 75% ethanol. The body length is measured from the anterior margin of the cephalic plate to the posterior end of the postpedal tergite. Type specimens are deposited in the School of Life Sciences, Hengshui University, Hengshui, China (**HUSLS**). The terminology of the external anatomy follows Bonato et al. (2010). Measurements are shown in millimeters (mm).

The following abbreviations are used in the text and the tables:

**T, TT** tergite, tergites;

**S, SS** sternite, sternites;

**C** coxa,

**r** trochanter,

**P** prefemur,

**F** femur,

**Ti** tibia,

**a** anterior,

**m** median,

**p** posterior,

**DaC spine** anterior dorsal spine of coxa.

## Taxonomy

### Australobius tracheoperspicuus sp. n.

Taxon classificationAnimaliaLithobiomorphaLithobiidae

http://zoobank.org/98D3BEF9-A361-40BF-BDE0-2B9400875C49

Figs 1–11 , Table 1

#### Material.

*Type material.* Holotype: male, Cave of the brickyard of Gaofeng village, Yancang Town, Yi-Hui-Miao Autonomous County of Weining , Bijie City, Guizhou Province, 26°54'30.95"N, 104°24'13.78"E, alt. 2430 m a.s.l., 19 V 2017, Huiming Chen leg. Paratypes: 1 ♀, 1 ♂, same data as holotype. *Other material.* 4♂♂(larvae), the Gaodiping Cave of Dashan village, shaanqiao street, Yi-Hui-Miao Autonomous County of Weining , Bijie City, Guizhou Province, 26°50'55.03"N, 104°17'08.81"E, alt. 2175 m a.s.l., 23 IV 2017, Huiming Chen leg.

#### Diagnosis.

Antennae with 26 articles, no ocelli, anterior margin of the coxosternite with 5+5 teeth, more or less developed, porodonts slender, between fourth and fifth outer teeth. Tergites without posterior triangular projections, trachea connected to the valve of the T III clearly visible from the dorsal side. Coxal pores 4–6. Tarsal articulation well defined on legs I–XV. No secondary sexual modifications on legs XIV and XV of male. Female gonopods with simple claw, 2+2 spurs. Male gonopods short and small blunt cone bulge, apically slightly sclerotized.

#### Description.

Body length 17.43 –19.24 mm, cephalic plate 1.76–1.93 mm long, 1.77–1.97 mm wide; the whole body pale yellow–brownish, tarsus II of all legs more darker, proximal parts of forcipules and the teeth of the anterior margin of the coxosternite brown, all claws of legs yellow–brown.

**Figures 1–12.**
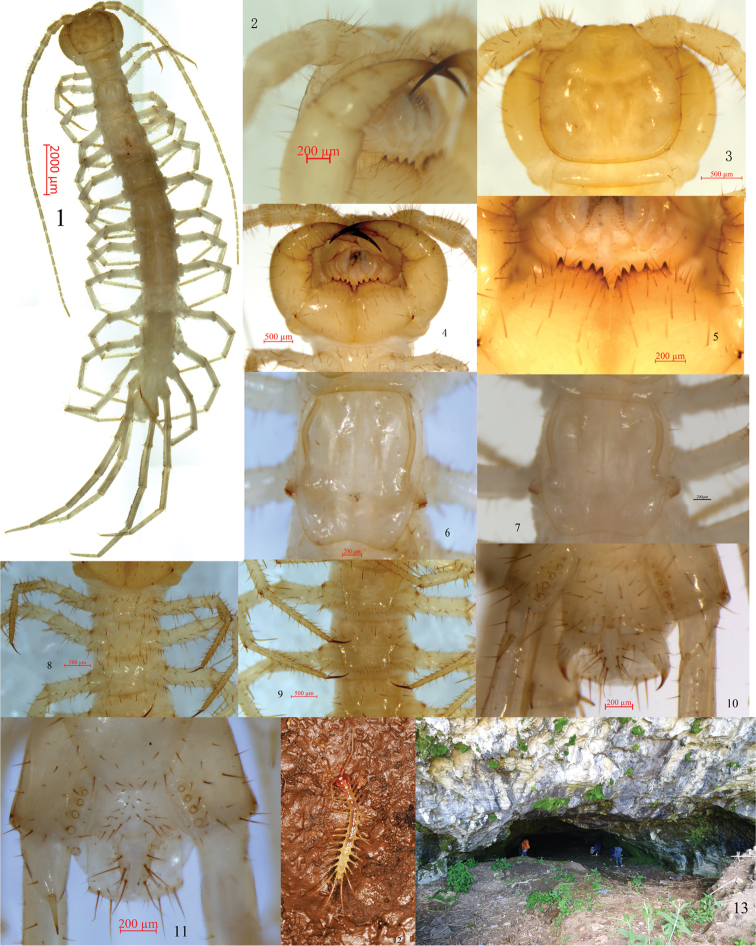
*Australobiustracheoperspicuus* sp. n. (holotype male **1–5, 7–9, 11–12** paratype female **6** and **10**) **1** Habitus, dorsal view **2** Tömösváry’s organ, lateral view **3** Cephalic plate, dorsal view **4** Cephalic plate, ventral view **5** Forcipular coxosternite, ventral view **6** T III of female **7** T III of male **8**SS I–V **9**SS VI and VII **10** Posterior segments and gonopods of female, ventral view **11** Posterior segments and gonopods in male, ventral view **12** Living specimen of *Australobiustracheoperspicuus* sp. n. **13** Cave of the brickyard of Gaofeng village.

Antennae with 26+26 articles; basal article slightly longer than wide, subsequent articles markedly longer than wide, distal article up to 2.5 times as long as wide. Abundant long setae on antennal surface, less so on basal articles, gradual increase in density of setae to about the fourth article, then more or less constant. Length of antenna 7.3–7.4 times width of cephalic plate, and often extending close to posterior edge of T XI (Figure 1).

No ocelli on each side of the cephalic plate. Tömösváry’s organ ovate, situated at anterolateral margin of the cephalic plate (Figure 2).

Cephalic plate smooth, convex, very slightly wider than long; tiny setae emerging from pores scattered very sparsely over whole surface; frontal marginal ridge with shallow anterior median furrow; from short to long setae scattered along marginal ridge of cephalic plate, there more setae close to the antenna; lateral marginal ridge discontinuous (Figure 3).

Forcipular coxosternite subtrapezoidal (Figure 4), anterior margin broad, external side lightly longer than internal side; median diastema moderately narrow, deeply V–shaped; anterior margin with 5+5 blunt teeth; porodonts slender, lying between the fourth and fifth outer teeth, and more closer to the fourth tooth, the innermost tooth more posterior, and the interdental distance gradually increases from the internal side to the external side (Figure 5); some short setae scattered on the ventral side of coxosternite; usually there are more setae near the dental margin.

All tergites with wrinkles, central backside slightly hunched, T I posterolaterally narrower than anterolaterally, generally trapeziform, narrower than cephalic plate, broader than T III. Trachea connected to the valve of the T III is clearly visible from the dorsal side (Figs 6–7). Posterior margin of T I slightly concave, posterior margin of TT III, V, VII, VIII, X, XII, XIV and XV concave. Marginal ridge of TT II , IV, VI, IX, X, XI, XII , XIII, XIV and XV bulging; lateral marginal ridge of all tergites continuous; all posterior angles generally rounded, without triangular projections; tiny setae scattered very sparsely over surface, more densely on anterior and posterior angles (Figure 1). All the tergites more longer than the congeneric species, T X is the longest, at most up to 1.3 times as long as wide.

Posterior side of sternites narrower than anterior one, generally trapeziform, comparatively smooth, long and thick setae emerging from pores scattered sparsely on surface, more setae on surface of the SS I–V (Figure 8), there are two irregular rows short and slightly thinker setae along the posterior margin of the SS VI and VII (Figure 9), few setae on surface of the following SS.

Legs long and slender, tarsal articulation well defined on legs I-IV; all legs with fairly long curved claws; anterior and posterior accessory spurs on legs I–XIII anterior accessory spurs moderately long and slender, forming an angle of about 45° with tarsal claws; posterior one slightly strong, forming an angle of about 30° with tarsal claws; no accessory spurs on legs XIV and XV. Comparatively long setae scattered very sparsely over surface of prefemur, femur, tibia and tarsus of legs I–XIII, more setae scattered on surface of tarsus; dorsal setae slightly longer than ventral, however, more setae in ventral; setae scattered on surface of legs XIV and XV clearly scarcer and short and fine than on other legs. Legs XIV and XV hardly thicker and stronger in both male and female, tarsus I about 6.3–6.6 times as long as wide in legs XV. Tarsus I of legs XV 5.0–5.6 times as long as wide in male, tarsus I of legs XV 5.0–5.6 times as long as wide in male. Leg plectrotaxy as in Table 1.

**Table 1.** Leg plectrotaxy of *Australobiustracheoperspicuus* sp. n.

Legs	Ventral side					Dorsal side				
	C	Tr	P	F	Ti	C	Tr	P	F	Ti
I–XII			mp	amp	am			amp	ap	ap
XIII			amp	amp	am	a		amp	ap	ap
XIV		m	amp	amp	a	a		amp	ap	ap
XV		m	amp	am	a	a		amp	a	

Coxal pores 4–6, most of them round, few ovate, 5-5-6-5 or 5-6-5-5 in female, 4-5-5-4 or 4-4-4-4 in male, coxal pore field set in a relatively shallow groove, fringe of coxal pore field with eminence, moderately long setae scattered sparsely over surface of eminence.

Female S XV anterolaterally broader than posterolaterally, generally trapeziform, posteromedially straight; sternite of genital segment usually well chitinised; posterior margin of genital sternite deeply concave between condyles of gonopods, except for a small, median bulge; long setae scattered over ventral surface of genital segment, regularly fringed, with longer setae along posterior margin. Gonopods: first article fairly broad, second moderately long and slender, coniform spurs in right, inner spur obviously larger than outer one (Figure 10); apical claw of third article simple, slender and sharp (Figure 11). Many long setae on surface of all segments of gonopods.

Male S XV posterolaterally narrower than anterolaterally, posterior edge straight, sparsely covered with long setae; sternite of genital segment smaller than in female, usually well sclerotised. Posterior margin quite deeply concave between gonopods, without a medial bulge; comparatively long setae scattered on ventral surface of genital segment, few slender setae near S XV, setae gradually increasing in density from anterior to posterior, gonopods short and small blunt cone bulge, apically slightly sclerotised (Figure 10).

#### Etymology.

The specific name refers to the trachea connected to the valve of the T III that is clearly visible from the dorsal side.

#### Habitat.

The specimens were collected on the limestone walls and bedrock floor of the cave.

#### Discussion.

The new species resembles *A.magnus* (Trozina>, 1894) from North-Western China in having the coxal pores numbering 6–7, no accessory spurs on legs XIV and XV, DaC spine present on legs XIII–XV, apical claw of female gonopods simple. However, the new species can be easily distinguished by the following characters: trachea connected to the valve of the TIII clearly visible from the dorsal side in new species instead of the trachea connected to the valve of the TIII is invisible from the dorsal side in *A.magnus*; absence of ocellus on each side of the cephalic plate vs. 8–9 ocelli in *A.magnus*; DaC spine being only present on the XIII^th^–XV^th^ legs in contrast to being present or absent on VIII^th^–X^th^ legs, present on X^th^ –XV^th^ legs in *A.magnus*.

On the other hand, several diagnostic features of the recently described Chinese species that are routinely used in the diagnosis of species of *Australobius* Chamberlin, 1920 are variable within the framework of the original description, perhaps the most conspicuous variation pertains to the number of teeth on the anterior margin of the coxosternite and the numbers of the antennal articles and the ocelli. For example, in the original description of the genus *Australobius*, the number of antennal articles is mostly 20, some species more than 24 articles, whereas in many species occurring in China it is higher (Table 2). The same is true for the number of the ocelli, which is few (e.g., 1+3–1+6) or in some species more than 8 in the original description of the genus, whereas in many species occurring in China it is higher (Table 2), and for the number of coxosternal teeth, which is at least 3+3 in the original description, whereas in many species occurring in China it is more variable (Table 2).

**Table 2.** Numbers of examined specimens, distribution and main morphological characters of the have known Chinese species of *Australobius* Chamberlin, 1920. Abbreviation: DaC spine, anterior spine of dorsal of coxa.

	* A. anamagnus *	* A. apicicornis *	* A. magnus *	* A. nodulus *	* A. tetrophthalmus *	*A.tracheoperspicuus* sp. n.
Original description	Ma et al. 2008a	Qin et al. 2014	Trotzina 1894	Ma et al. 2008b	Loksa 1960	This paper
Specimens examined	2♀1♂	3♀2♂	1♀	4♀2♂	1♂	1♀2♂(4♂larvae)
Other sources	no	no	Eason 1997, Dyachkov 2017	no	Eason 1978	no
Specimens examined	no	no	13♀6♂, 34♀8♂	no	1♂	no
Distribution	Qinghai-Tibet Plateau China (Tibet)	Qinghai-Tibet Plateau China (Sichuan)	Qinghai-Tibet Plateau China (Tibet), Kirghizia and Kazakhstan	Qinghai-Tibet Plateau, China (Tibet)	China S (Guangxi)	China S (Guizhou)
Body length (mm)	15.9 – 26.6	17.6 – 22.5	16.0 – 30.0	17.1 – 22.1	19.0	17.4 –19.2
Number of antennal articles	26+26, rarely 25+26	24+24	25+25 – 30+30	31+31 – 33+33	29	26+26
Number and arrangement of ocelli	10, in 2 rows	7 – 9, in 2 rows	8 – 9, in 2 rows	9 – 11, in 2 rows	4, in 2 rows	None
Tömösváry's organ	Nearly round, smaller than adjoining ocelli	Round, smaller than adjacent ocellus	Round, smaller than adjacent ocellus	Smaller than adjoining ocelli	Ovate, larger than adjoining ocelli	Ovate
Number and arrangement of coxosternal teeth	3+3, 3+4, 4+4	8+6, 5+5, 6+6, 5+6, Roughly triangular	2+2 – 7+7, few 3+4, 6+7, small, blunt	6+6 or 6+5, small and of	5+5, small blunt	5+5
Porodont	Comparatively thick and strong, situated between outer two teeth, few between second and third	Absent	Short and pointed, situated between outer two teeth, or between second and third	Situated between outer third and fourth teeth, rarely between second and third teeth	Not reported	slender, lying between the fourth and fifth outer teeth, and more closer to the fourth tooth
Number of coxal pores	4 – 9 Females: 5-6-7-6, 5-7-7-7, 6-7-7-6,	5 – 8, usually 6-6-6-6, 6-6-6-5, 8-8-8-8, 6-7-7-6, 6-7-7-7, 6-7-8-7, 6-7-9-7	3 – 7, rarely 8	4 – 7 arranged into an irregular row, 5-6-6-5, 4-5-5-5, 6-7-7-6, 6-7-7-5	3-3-3-3	4 – 6, 5-5-6-5 or 5-6-5-5 in female, 4-5-5-4 or 4-4-4-4 in male
DaC spine	On XII^th^ (present or absent) –XV^th^ legs	On XIII^th^ –XV^th^ legs	On VIII^th^ –X^th^ present or absent, on XI^th^ –XV 15^th^ legs present	On VII^th^ –XV^th^ legs	Absent	On XIII^th^ –XV^th^ legs
legs XIV^th^ accessory spur	Absent	Present	Absent	Absent	Not reported	Absent
legs XV^th^ accessory spur	Absent	Absent	Absent	Absent	Not reported	Absent
Number and shape of spurs on female gonopods	3+3, 3+4, 4+4 moderately small, inner coniform spurs, inner one much smaller	3+3 or 4+4 coniform spurs, ones smaller than outer spurs	2+2 – 4+4, rarely 4+5	2+2 or 4+4 moderately small, coniform spurs, inner spur clearly smaller than outer one	Not reported	2+2 moderately long and slender, coniform spurs, inner spur larger than outer one,
Apical claw of female gonopods and lateral denticles	Broad, simple	Simple	Simple	Broad, simple	Not reported	simple, slender and sharp
Male gonopods	A small hemispherical protuberance, with a single long seta, distal region slightly sclerotised	small, indistinct swellings, with one or two long setae	small, spherical	Small hemispheroid protuberance, with3 – 4 long setae, apically slightly sclerotized	Not reported	small small blunt cone bulge, apically slightly sclerotised

To assist in the identification of the Chinese species of *Australobius*, numbers of examined specimens, distribution and main morphological characters of the known species of this genus in China is presented (Table 2) and key to the known Chinese species of the genus is presented, these characters are specific only to adults of the taxa occurring in China.

##### Key to the known Chinese species of the genus *Australobius* Chamberlin, 1920

**Table:** 

1	No ocelli on each side of cephalic plate	***A.tracheoperspicuus* sp. n.**
–	At least four ocelli on each side of cephalic plate	**2**
2	Four ocelli on each side of cephalic plate, Tömösváry’s organ larger than adjacent ocelli	***A.tetrophthalmus* (Loksa, 1960)**
–	More than seven ocelli on each side of cephalic plate, Tömösváry’s organ smaller than adjacent ocelli	**3**
3	No prodonts	***A.apicicornis* Qin, Lin, Zhao, Li, Xie, Ma, Su & Zhang, 2014**
–	Porodonts present	**4**
4	Large posterior tergites wrinkled; bulge present on terminal part of tarsus	***A.magnus* (Trozina, 1894)**
–	Large posterior tergites smooth; no bulge on the terminal part of tarsus	**5**
5	Antenna with at most 26 articles and 2+2 or 4+4 forcipular coxosternal teeth	***A.anamagnus* Ma, Song & Zhu, 2008**
–	Antenna with at least 31 articles and at most 6+6 forcipular coxosternal teeth	***A.nodulus* Ma, Song & Zhu, 2008**

## Supplementary Material

XML Treatment for Australobius tracheoperspicuus
